# Elongated Polyproline Motifs Facilitate Enamel Evolution through Matrix Subunit Compaction

**DOI:** 10.1371/journal.pbio.1000262

**Published:** 2009-12-22

**Authors:** Tianquan Jin, Yoshihiro Ito, Xianghong Luan, Smit Dangaria, Cameron Walker, Michael Allen, Ashok Kulkarni, Carolyn Gibson, Richard Braatz, Xiubei Liao, Thomas G. H. Diekwisch

**Affiliations:** 1 Brodie Laboratory for Craniofacial Genetics, University of Illinois at Chicago College of Dentistry, Chicago, Illinois, United States of America; 2 University of Chicago, Chicago, Illinois, United States of America; 3 National Institutes of Health, Functional Genomics Unit, Bethesda, Maryland, United States of America; 4 University of Pennsylvania, Philadelphia, Pennsylvania, United States of America; 5 Department of Chemical and Biomolecular Engineering, University of Illinois at Urbana-Champaign, Urbana-Champaign, Illinois, United States of America; 6 Department of Biochemistry and Molecular Biology, University of Illinois at Chicago, Chicago, Illinois, United States of America; University of Leeds, United Kingdom

## Abstract

Vertebrate body designs rely on hydroxyapatite as the principal mineral component of relatively light-weight, articulated endoskeletons and sophisticated tooth-bearing jaws, facilitating rapid movement and efficient predation. Biological mineralization and skeletal growth are frequently accomplished through proteins containing polyproline repeat elements. Through their well-defined yet mobile and flexible structure polyproline-rich proteins control mineral shape and contribute many other biological functions including Alzheimer's amyloid aggregation and prolamine plant storage. In the present study we have hypothesized that polyproline repeat proteins exert their control over biological events such as mineral growth, plaque aggregation, or viscous adhesion by altering the length of their central repeat domain, resulting in dramatic changes in supramolecular assembly dimensions. In order to test our hypothesis, we have used the vertebrate mineralization protein amelogenin as an exemplar and determined the biological effect of the four-fold increased polyproline tandem repeat length in the amphibian/mammalian transition. To study the effect of polyproline repeat length on matrix assembly, protein structure, and apatite crystal growth, we have measured supramolecular assembly dimensions in various vertebrates using atomic force microscopy, tested the effect of protein assemblies on crystal growth by electron microscopy, generated a transgenic mouse model to examine the effect of an abbreviated polyproline sequence on crystal growth, and determined the structure of polyproline repeat elements using 3D NMR. Our study shows that an increase in PXX/PXQ tandem repeat motif length results (i) in a compaction of protein matrix subunit dimensions, (ii) reduced conformational variability, (iii) an increase in polyproline II helices, and (iv) promotion of apatite crystal length. Together, these findings establish a direct relationship between polyproline tandem repeat fragment assemblies and the evolution and the design of vertebrate mineralized tissue microstructures. Our findings reveal that in the greater context of chordate evolution, the biological control of apatite growth by polyproline-based matrix assemblies provides a molecular basis for the evolution of the vertebrate body plan.

## Abstract 2

How does proline-repeat motif length in the proteins of teeth and bones relate to the evolution of vertebrates? Counterintuitively, longer repeat stretches are associated with smaller aggregated subunits within a supramolecular matrix, resulting in enhanced crystal length in mammalian versus amphibian tooth enamel.

## Author Summary

The microstructure of vertebrate bones and teeth is controlled by polyproline-rich protein matrices (such as amelogenin) that serve as a scaffold to control the assembly of biological apatites. In tooth enamel, amphibians have large amelogenin subunits and thin enamel while mammals have smaller amelogenin subunits in tandem with elongated crystals and complex prismatic organization. Using specific peptides and frog amelogenin overexpressed in mice, we confirmed the effect of the length of the elongated polyproline repeat on reduced matrix subunit dimensions and enhanced apatite crystal length. Three-dimensional structures solved by NMR (nuclear magnetic resonance) and surface modeling algorithms indicate that elongated polyproline repeat stretches in amelogenins affect the dimensions of the supramolecular matrix through an increase in polyproline II helices, resulting in a compaction of supramolecular subunit dimensions. We propose that the availability of readily shaped apatites and innovative mechanisms based on amelogenin-repeat motifsthat compartmentalize and shape biological minerals was essential for the rise of early vertebrates, enabling the manufacture of strong teeth and backbones that might have given vertebrates a decisive survival advantage in the competition for food and in the sophistication of locomotion.

## Introduction

Proline-rich regions occur in a wide variety of functionally significant proteins, including mucins, snow flea antifreeze proteins, prolamine storage proteins, pancreatic polypeptide hormones, neuropeptides, Alzheimer amyloid, prion proteins, and tooth enamel proteins [1],[2]. Many proline-rich proteins contain repetitive motifs and adopt left-handed polyproline II helical conformations (PPII) [3],[4]. These PPII helices are more mobile than other periodic structures, e.g. α-helices or β-sheets [5], but nevertheless exhibit well-defined molecular backbone conformation due to the rigidity of the proline ring. The well-defined yet mobile and flexible structure of polyprolines has led to the hypothesis that such proteins may function as mineral-binding domains, protein-protein docking domains, or internal molecular spacers during the formation of biological minerals and other biocomposites [6]. Remarkably, proline-rich, tripeptide tandem repeat proteins participate on all levels of biological mineralization and include members as diverse as the *Haliotis rufescens* protein Lustrin A involved in the extracellular deposition of shell and pearl, the *Strongylocentrotus purpuratus* protein SM50 contributing to the mineralization of sea urchin teeth and spicules from magnesium calcite and protodolomite, as well as vertebrate collagen I and the tetrapod tooth enamel protein amelogenin [6],[7].

The rise of vertebrates coincides with the emergence of revolutionary body designs that rely on hydroxyapatite as the principal mineral component of bones and teeth [8]. Vertebrates use apatites to form relatively light-weight, articulated endoskeletons and sophisticated tooth-bearing jaws, facilitating rapid movement and efficient predation. A large degree of flexibility (Greek: apatite = deceit) allows apatites to be readily shaped by proline-rich proteins such as collagen I and the tooth enamel protein amelogenin.

Apatite mineral growth and habit in vertebrate enamel are controlled by a unique proline-rich protein, amelogenin, which forms the majority of the developing enamel matrix (about 95%) [9]. A recent study has shed new light on the organization of this relatively unstructured protein [10]. Other studies have indicated that amelogenin self-assembly might be mediated by a complementary relationship between the hydrophobic and PPII helical regions [11],[12]. Difficulties in obtaining protein crystals suitable for X-ray crystallography have prompted a series of studies using circular dichroism (CD), NMR, Raman spectroscopy, and molecular modeling studies [13]. Earlier CD, FTIR, and Raman spectroscopy experiments suggested mixed β-sheet/β-turn/helix and random coil structures [7],[14],[15] with extended β-spiral/poly-L-proline type II (PPII) helical structures in the midsection of amelogenin [13]. The importance of the amelogenin N-terminus for amelogenin self-assembly has been confirmed by yeast-two-hybrid studies and biochemical analysis of the two serine residues in positions 16 and 25 [16],[17]. Based on solid state NMR data, the amelogenin carboxy-terminal domain appears to be oriented next to the hydroxyapatite crystal surface [18]. Loss of the carboxy-terminus as it occurs during amelogenin proteolytic processing has been associated with a reduced affinity to hydroxyapatite and a reduction in the ability to inhibit crystal growth [19],[20]. Recent crystal growth studies suggest that the carboxy-terminus is important for the alignment of crystals into parallel arrays while the remainder of the molecule plays a role in the inhibition of crystals growth [21].

With the emergence of prismatic enamel in mammals, the length of amelogenin polyproline tri-peptide repeats increases significantly, suggesting that the augmentation of amelogenin proline-rich regions is governed by evolutionary trends. The augmentation in polyproline repeat length occurs within the proline-rich amelogenin peptide (PRAP, i.e. the region from AA46–AA166), which is comprised of an evolutionary “hotspot” containing a series of PXX tandem repeats [22],[23]. The rapid evolution of the PRAP from amphibian to mammals was primarily accomplished by insertions of PXX tripeptide motifs [24], with PXQ as the most frequent tripeptide sequence element. In these extended polyproline repeat structures, both proline and glutamine cause structural rigidity of the newly added tripeptide complexes [25].

The unique occurrence of elongated polyproline stretches and amelogenin protein assembly in the evolution of the vertebrate dentition prompted us to ask the question of whether repeat length and self-assembly dimensions were related and whether there was any association between polyproline repeat motif length and structural changes in mineral shape and matrix organization. In order to ask this question, we compared polyproline repeat length and amelogenin nanosphere dimensions between vertebrates and generated a number of biomimetic peptides. We then tested the relationship between mammalian and amphibian polyproline repeat length, nanosphere assembly, and crystal growth in a frog amelogenin overexpressing mouse model. Finally, we determined the 3D NMR structure of the amelogenin repeat region to identify unique structural motifs explaining the correlation between amelogenin self-assembly and polyproline repeat length. In the present study we are demonstrating that the unique ability of polyproline motifs to shape biological minerals lies in their ability to alter protein matrix self-assembly. We are arguing that in the greater context of chordate evolution, the biological control of apatite growth by polyproline-based matrix assemblies provides a molecular basis for the evolution of the vertebrate body plan.

## Results/Discussion

### Non-Mammalian Amelogenins Contain Fewer Proline Tripeptide Repeat Sequences Than Mammals

**Figure 1:**
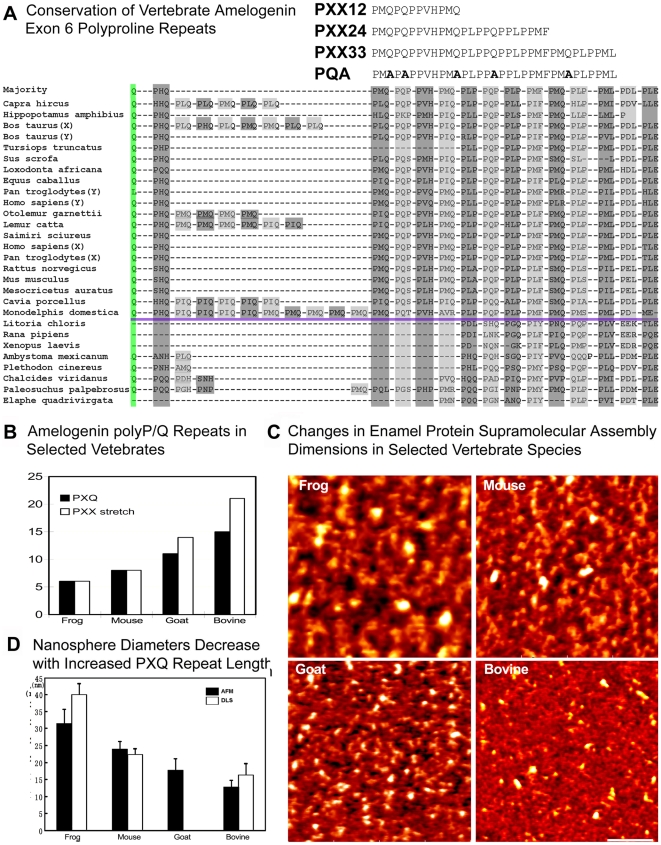
Polyproline repeats in the evolution of bilaterian mineralization systems. (A) Conservation and evolution of the tooth enamel protein amelogenin. Note the high conservation of PXX repeat elements among mammals (e.g., Homo, Mus), reptiles (e.g., Elaphe, Paleosuchus), and amphibians (e.g., Xenopus, Rana) (light and dark grey shaded areas). The conserved PXX majority repeat region served as a blueprint for our designer peptides PXX12, PXX24, and PXX33. In addition, a fourth designer peptide (PQA) was synthesized in which glutamine was replaced by alanine. (B) Increase in PXQ PXX repeat region length from amphibians to rodents and then once more to ruminants. (C) Atomic force microscopy (AFM) images of extracted enamel proteins from diverse vertebrate species. Note the increase in aggregate (nanosphere) dimensions from bovine to goat to mouse and to frog. (D) Highly significant differences between average species-specific enamel protein nanosphere diameters based on statistical evaluation of AFM images and dynamic light scattering (DLS). Bovine nanospheres were less than half the size of frog counterparts.

PXX repeat element organization is highly conserved between mammals (e.g., Homo, Mus), reptiles (e.g., Elaphe, Paleosuchus), and amphibians (e.g., Xenopus, Rana) (Figure 1A). There were fewer PXX repeat elements in amphibians, while several mammalian species (e.g., ruminants and marsupials) featured PXX repeat numbers exceeding those found in humans or mice (Figure 1A, 1B). A comparison with known amelogenin sequences indicated that the number of PXX repeats in the frog *Rana pipiens* was significantly shorter than the PXX repeat number in mice, goats, or steers (Figure 1B). This comparison indicates a potential trend toward increased polyproline repeat length with increasingly sophisticated enamel structures in vertebrates.

### Species with Long Amelogenin PXX Repeat Stretches Have Smaller Nanospheres Than Those with Short Polyproline Repeat Regions

In order to determine whether changes in PXX repeat length were associated with changes in supramolecular enamel protein assembly dimensions in nature, we compared repeat length and supramolecular assembly dimensions from four selected vertebrate species using atomic force microscopy (AFM) and dynamic light scattering (DLS; Figure 1B–D). Native enamel matrix proteins from frog (*Rana pipiens*), mouse (*Mus musculus*), goat (*Capra hircus*), and bovine (*Bos taurus*) were chosen to represent increasing PXX repeat length in vertebrates (Figure 1B). Both the AFM and the DLS analysis demonstrated that enamel protein supramolecular assembly dimensions gradually decreased by 60% from frog to bovine, while PXX length gradually increased by 250% (Figure 1B–D), suggesting an inverse correlation between polyproline repeat length and enamel protein 3D-assembly dimensions in the evolution of vertebrate enamel proteins.

### PXX Designer Peptide Length Determines Apatite Crystal Growth

**Figure 2:**
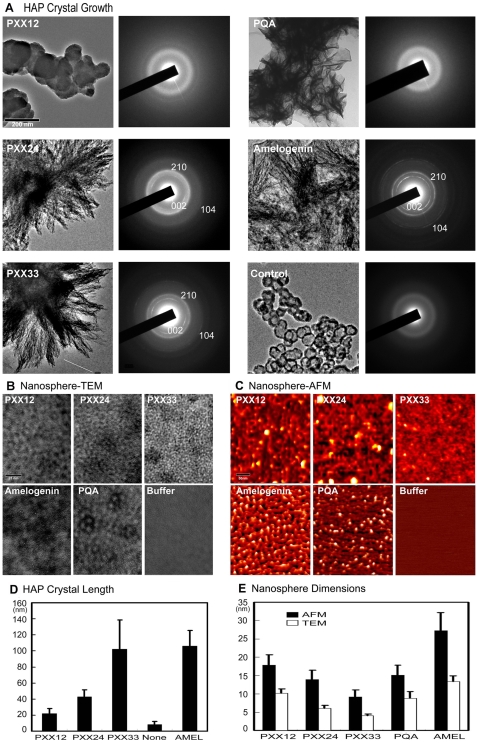
Hydroxyapatite crystal growth control and self-assembly of polyproline designer peptides. (A and D) Effect of polyproline designer peptides on hydroxyapatite (HAP) crystal growth. Increasing length of designer peptides from PXX12 to PXX33 resulted in elongated HAP crystal (A). HAP crystals grown with PXX33 were similar in length to those grown with full-length amelogenin. The PQA glutamine/alanine replacement peptide resulted in thin plates unlike the needles grown with PXX repeat peptides. HAP control solutions incubated without protein formed spherical deposits. Crystal dimensions are documented in (D). Bar = 200 nm. Electron diffraction analysis of PXX24, PXX33, and amelogenin treated samples resulted in sharp and distinct reflection rings in the 002 and 210 planes and an additional faint ring in the 104 plane, similar to those found in developing enamel crystals. Control crystals and those grown in the presence of PXX12 and PQA only showed faint diffraction patterns. (B, C, and E) Effect of polyproline designer peptide length on supramolecular matrix assemblies. Both TEM and AFM analyses indicated that matrix subunit dimensions were significantly reduced with increasing designer peptide length (from PXX12 to PXX33). Full-length amelogenins formed sizable nanospheres measuring 27 nm by AFM and 13 nm by TEM in diameter. Matrix dimensions and assembly patterns of the PQA glutamine/alanine replacement peptide were significantly different from their PXX33 counterparts. Buffer control solutions did not assemble into nanosphere-like structures. All measurements in this study were statistically evaluated and displayed using standard deviation (s.d.). Bar (TEM) = 25 nm, (AFM) = 50 nm.

In order to determine the effect of polyproline designer peptides of increasing length (Figure 1A) on apatite crystal growth, crystals were grown in the presence of PXX polyproline designer peptides or amelogenins. Addition of PXX designer peptides to the crystallization solution resulted in the formation of needle-shaped crystallites, and longer PXX repeat motifs corresponded with increased crystal length (PXX12: 21.6±6.5 nm, PXX24: 42.9±8.5 nm, PXX33: 102.1±36.3 nm). Addition of recombinant full-length amelogenin (rM180) resulted in the formation of elongated crystals of 106.2±19.3 nm length. Hydroxyapatite crystals grown without any addition of protein measured 8.2±3.9 nm in length while the 33 mer glutamine/alanine replacement polypeptide PQA yielded flake-like particles with broad diffraction rings (Figure 2A, 2D). There were distinct differences in diffraction patterns between crystals grown under the control of various additives (Figure 2A). The control only showed diffuse diffraction patterns indicative of amorphous calcium phosphate. Both the PXX12 and the PQA sample also revealed only diffuse diffraction rings. The PXX24 treated sample featured a preferred orientation in the 002 plane and a diffuse reflection ring in the 210 plane. Both the PXX33 and the amelogenin treated samples displayed sharp rings in both the 002 and the 210 plane. There was also a very faint reflection ring in the 104 plane of the PXX24, the PXX33, and the amelogenin samples. These findings demonstrate that PXX designer peptides with increased length yield significantly longer apatite crystals with diffraction patterns similar to those of developing enamel apatite crystals [29]. Specifically, 12 mer polyproline repeat stretches were associated with amorphous apatite while stretches of 24 mer and above featured a crystalline mineral phase. They also document that PXX polyproline peptides alone exert a profound control on apatite crystal growth. We interpret these results to indicate that the polyproline backbone of elongated PXX repeat peptides enhances protein matrix structural rigidity, resulting in an inhibition of epitaxial apatite crystal growth on a- and b-axis surfaces while promoting apatite crystal growth in c-axis direction.

### PXX Designer Peptide Length Determines Supramolecular Matrix Assembly

In order to further understand the mechanisms by which polyproline repeat peptides affect crystal growth, we decided to test the effect of polypeptide length on protein matrix organization. In previous studies we demonstrated that the extracellular protein matrix of developing tooth enamel provides a complex supramolecular biomineralization template that directly controls enamel crystal formation [9],[26]. Here we once more used our PXX12, PXX24, and PXX33 peptides to ask the question whether the length of these polypeptides affects organic matrix organization. In order to address this question, protein assemblies on coated carbon grids were studied using transmission electron micrographs (TEM) (Figure 2B). In addition, AFMs of proteins in solution were generated (Figure 2C). Our results demonstrated that protein matrix nanosphere diameters were 17.9±2.7 nm^AFM^ (10.1±1.2 nm^TEM^) for PXX12, 13.9±2.6 nm^AFM^ (6.1±0.8 nm^TEM^) for PXX24, and 9.2±1.9 nm^AFM^ (4.0±0.5 nm^TEM^) for PXX33. Nanosphere diameters of the two controls were 27.3±4.6 nm^AFM^ (13.3±1.5 nm^TEM^) for the recombinant full-length mouse amelogenin control (rM180) and 15.1±2.8 nm^AFM^ (8.8±1.9 nm^TEM^) for the 33 mer glutamine/alanine replacement polypeptide PQA (Figure 2E). In average, TEM nanosphere dimensions were about 50% of their AFM counterparts, a difference that might be explained by the differences in sample preparation between the dissolved protein used for AFM and the dried TEM sample. Sample buffers did not yield any significant substructures. Both AFM and TEM data demonstrated that nanosphere diameters decreased with increasing peptide length, i.e. PXX33 nanospheres measured about half the size of PXX12 nanospheres and were double as densely packed. This apparent readiness of extended PPII helices to assume a high level of compaction might be explained by a dramatic reduction in conformational entropy in such an assembly [27].

A comparison of mouse and frog amelogenin sequences indicated that the number of prolines was 34 and 27 in mouse and *Rana pipiens* PRAPs, respectively, and that the major difference between mouse and frog amelogenins was a 33% higher number of PXX repeat motifs in mice versus frogs (Figure 1A, 1B). Glutamine is the second most likely residue to appear in a PPII helix segment (second to proline) [4] and thus a likely partner to interact with prolines in the function of PPII helices. We were thus interested in testing the effect of glutamines on the macromolecular assembly of polyprolines. Remarkably, when the 5 glutamines in PXX33 were exchanged with alanine substitutes (PQA peptide), nanosphere diameters about doubled and electron density distribution on micrographs was drastically altered. The 33 mer glutamine/alanine replacement polypeptide PQA did not yield any HAP crystals of measurable length (Figure 2A). The loss of HAP crystal extensions underscores the importance of glutamine insertions in the overlying polyproline repeat sequence for crystal growth. The glutamine substitutions with alanine, effectively reversing the effect of extended polyproline macromolecular compaction found in PXX stretches, also indicate that glutamines play a pivotal role in the compaction of PPII helices as they occur in many biological systems, including biominerals.

### Disturbed Enamel Formation and Greater Supramolecular Enamel Matrix Dimensions in Frog Amelogenin Expressing Mice

**Figure 3:**
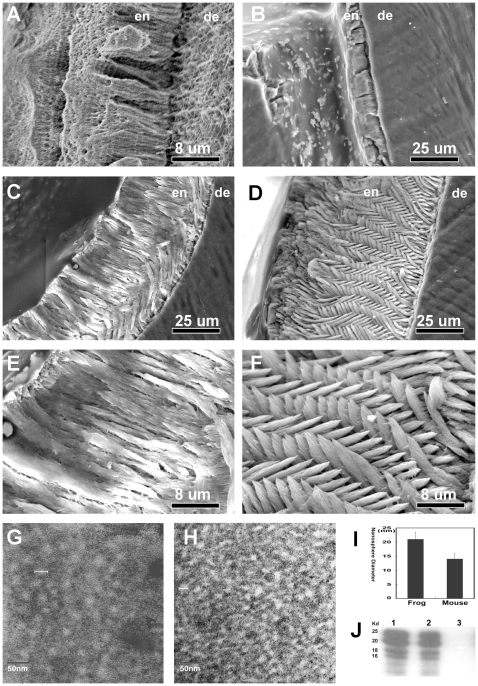
Differences in enamel prism formation and enamel matrix structure between molars from wild-type and frog amelogenin overexpressing mice. Note the prism-less organization of frog enamel (A) and the complete loss of structured enamel in the amelogenin null mice (B). In these scanning electron micrographs, enamel (en) and dentin (de) were labeled for orientation purposes. Frog amelogenin overexpressing offspring crossed with amelogenin null mice (fAmel-x-null) demonstrated reduced enamel thickness (C/E versus D/F) and grossly altered enamel prism structure with fused individual crystallites, especially in the coronal half of the transgenic enamel layer (C/E versus D/F). Notably, fAmel-x-null mouse enamel lacked the regularly intercrossed prism pattern found in wild-type mouse molars (C/E versus D/F). In order to compare the effect of frog amelogenins on enamel matrix structure when compared to mouse amelogenins, enamel matrix nanosphere diameters were determined on transmission electron micrographs from the enamel matrix of developing mouse first mandibular molars (G–I). Subunits measured 20.9±2.6 nm in frog enamel chimera and 13.9±1.9 nm in their wild-type controls (G–I). Enamel matrix nanosphere dimensions in fAmel-x-null enamel exceeded those of regular mouse enamel by about 50%. (G–I) Cleavage patterns of extracted mouse enamel proteins from fAmel-x-null mice (lane 1) and wild-type mice (lane 2) were almost identical (J), while there was no amelogenin detected in amelogenin null mice (lane 3). Equal protein loads were subjected to an antibody against recombinant amelogenin for Western blotting (J).

While hydroxyapatite crystals of mammalian enamel are organized into tightly packed rods (prisms) [26],[28],[29], this regular organization in enamel prisms is largely absent in amphibians and reptilians [30]. On a molecular level, the emergence of prismatic enamel organization during the amphibian/reptile to mammal transition has been paralleled by a significant increase in amelogenin PXX repeat length (Figure 1A) [31]–[36]. We have thus hypothesized that the unique arrangement of elongated mammalian apatite crystals into prisms is a result of PXX repeat length in the PRAP. In order to examine the effect of a shortened polyproline repeat amelogenin on mouse enamel formation, *Rana pipiens* amelogenin expressing mice (fAmel-x-null mice) were generated by cross-breeding amelogenin null mice with *Rana pipiens* amelogenin transgenic overexpressors to prevent the normal mouse amelogenin background from interfering with the transgenic phenotype. In this model, frog amelogenins were cleaved in a very similar fashion to their murine counterparts (Figure 3J). Enamel of first mandibular molar from five amelogenin null, fAmel-x-null, and wild-type mice each was analyzed and compared between groups. The phenotype in frog amelogenin overexpressors that were not crossed with null mice (fAmel) was less severe and thus not used for further analysis. Amelogenin null mice, which were used as a control, only featured a rudimentary mineral deposit on the surface of the underlying dentin (Figure 3B). Comparison between first mandibular molar enamel of fAmel-x-null mice and wild-type controls demonstrated 50.3% reduced enamel thickness and grossly altered enamel prism structure including massive patches of fused crystallites, especially in the coronal half of the fAmel-x-null enamel layer (Figure 3C, 3E versus 3D, 3F). We attribute this change in enamel prism pattern to a drastic reduction of PXX repeat stretches in the frog amelogenin compared to its mouse counterpart, resulting in an impairment of protein assemblies to coat and package individual elongated crystallites. The prism-less organization of fAmel-x-null enamel also somewhat resembled the prism-less structure of frog enamel (Figure 3E versus Figure 3A), suggesting that amelogenins with elongated polyproline stretches might be one requirement for prismatic enamel. Enamel matrix nanosphere diameters were 20.9±2.6 nm in fAmel-x-null mice and 14.0±1.9 nm in their wild-type controls (Figure 3G–3I). As a result, enamel matrix nanosphere dimensions in fAmel-x-null mice exceeded those of controls by 50.1%. These findings are corroborated by DLS-based comparisons demonstrating significantly larger frog amelogenin nanospheres compared to mouse amelogenin nanospheres (Figure 1D). Together, these studies document that the presence of increased length of polyproline repeat stretches in mammalian amelogenins is associated with both reduced macromolecular assembly dimensions and sophisticated mammalian enamel crystal/prism structure. Alterations in prism organization as seen as a result of elongated polyproline stretches may also entail additional mechanisms not highlighted in the present study. Cellular effects such as changes in cell movement pattern or in ameloblast morphology might also contribute to the phenotype observed and other portions of the amelogenin molecule are likely to be involved in amelogenin nanosphere assembly as well, even though their exact contributions remain to be established.

### Elongated Amelogenin-Based PXX Repeat Peptides Feature Left-Handed PPII Helices, Reduced Structural Variability, and Reduced Electrostatic Potential

In order to identify unique structural features and to explore the effect of repeat motif elongation on enamel matrix organization, we have performed a series of structural analyses based on the longest PRAP-derived designer peptide PXX33. In the absence of amelogenin X-ray crystallography data, CD, FTIR, and Raman spectroscopy studies have suggested mixed β-sheet/β-turn/helix and random coil structures [7],[37] with extended β-spiral/poly-L-proline type II (PPII) helical structures in the PRAP [10]–[12]. In order to determine structural features of the PRAP-derived PXX33 polypeptide, NMR analysis was performed and chemical shifts of 20 out of 33 amino acid residues of the PXX33 peptide were completely or partially assigned (Table S1), providing a basis for subsequent NOE (Nuclear Overhauser Effect) analysis. Using Nuclear Overhauser Effect Spectroscopy (NOESY), a total of 151 (NOE) definitive restricts were obtained, including 20 intra-amino acid residues and 100 neighbor amino acids [dN(i, i+1)], 24 dN(i, i+2), 5 dN(i, i+3), and 2 dN(i, i+4) NOEs. No long distance NOEs were detected (Table S2). Analysis of chemical shifts and NOE patterns did not reveal any typical α-helix or β-strand secondary structures.

**Figure 4:**
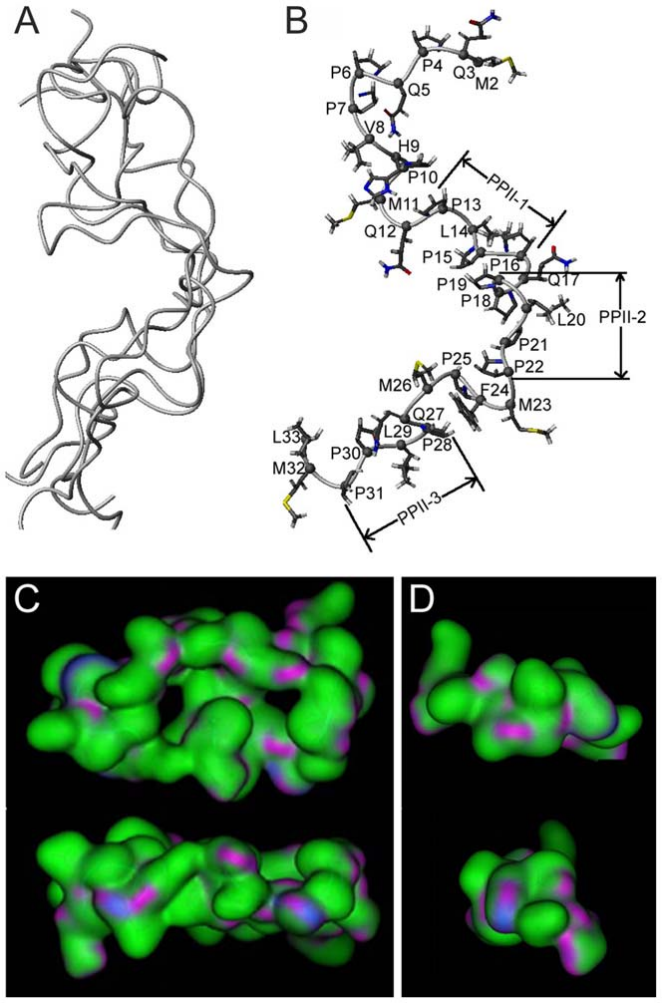
PXX33 atomic structure derived from solution NMR. (A) The five lowest energy structures selected from 200 calculated structures represented in ribbon form. While the overall structure was similar between all five conformations, there was a greater variability at the N-terminal PXX12 region. (B) Backbone ribbon representation and side chain heteroatom representation of one PXX33 lowest energy structure. Three polyproline II helix regions—P13–P16 (PPII-1), P19–P22 (PPII-2), and P28–P31 (PPII-3) —are labeled. (C–D) Increase in surface area in larger PXX33 polypeptides (C) versus PXX12 polypeptides (D), resulting in increased interaction, van der Waals attraction, and denser aggregates.

In order to calculate and analyze the three-dimensional structure of the PXX33 peptide at atomic resolution, NOE constrains were entered into the DYANA software package to calculate a total of 200 candidate structures. There was a fairly high backbone root mean square deviation (RMSD) of 7.51±1.51 Å and a heavy atom RMSD of 8.56±1.54 Å between the 200 candidate structures investigated. The absence of long distance NOEs suggest that PXX33 forms extended structures in aqueous solution while the high RMSD values imply a lack of well-defined conformations. In order to illustrate the similarities and slight variation between individual candidate structures, five structures representing lowest energy conformations were plotted together and superimposed using the MolMol software (Figure 4A). Our analysis revealed significantly higher structural variability at the PXX33 N-terminus representing the PXX12 polypeptide (Figure 4A). The high structural variability between various conformations in the PXX12 region might be one of the reasons for the larger size and irregular boundaries of the PXX12 nanospheres compared to their PXX24 and PXX33 counterparts. Further analysis of individual lowest energy conformations revealed three left-handed extended PPII helices (PPII-1, P13–P16; PPII-2, P19–P22; PPII-3, P28–P31) (Figure 4B), which were identified using the following criteria: (i) left-handedness, (ii) 3 amino acid residues per turn, and (iii) 3.1 Å per residue advance (9.3 Å per turn). Individual PPII turns measured 8.8 Å (PPII-1), 9.3 Å (PPII-2), and 9.45 Å (PPII-3). These were four residue-length PPII helices in which the proline rings at the positions i and i+3 were oriented in the same direction [1],[38]. The presence of three PPII helices in the amino acid region 13–33 and the absence of PPII helices in the PXX12 stretch might be another reason for the enormous compaction observed in PXX33 supramolecular assemblies compared to PXX12 and PXX24 counterparts as PPII helices have been associated with unusual structural compactness [38].

Several other factors may also explain the dramatic compaction of PXX33 supramolecular assemblies compared to aggregates formed by shorter polypeptides. It is widely accepted that hydrophobic free energy is a major force driving peptide-peptide interactions [39]. The surface of the larger PXX33 peptide (Figure 4C) is at least as hydrophobic as the smaller PXX12 peptide (Figure 4D), and its increased surface area provides more contacts for interaction and more van der Waals attraction. An increased attraction between the larger peptides would be consistent with the formation of aggregates with higher density, which was observed experimentally. Another factor contributing to the reduced size of PXX33 assemblies might be their reduced mobility, especially in light of the flexibility of polyproline structures in solution. The mean thermal velocity of a peptide due to Brownian motion is inversely proportional to the square root of its mass [40], resulting in smaller peptides in an aggregate to impact each other with higher frequency, which in turn would weaken the strength of the aggregate and reduce its density.

### Perspective

Already Hellenistic culture knew of the enormous adaptability, variability, and flexibility of apatites, using the word απαταω (to deceive) in reference to the similarities between apatites and other minerals. Five hundred million years earlier, during the Ordovician, the first vertebrates took advantage of these versatile minerals as building blocks for newly designed endoskeletal backbones and teeth. The incorporation of apatites into early vertebrate body designs was likely facilitated by SPARC and SPARCL1 as ancestors of SCPP mineralization proteins that arose at the same time by tandem duplication [41]. These early SPARC proteins might have served as templates for insertion-based repeat length expansion as it is associated with the generation of intrinsically unstructured proteins [42],[43]. The significant variation in enamel structure and polyproline repeat length among mammals (e.g. between ruminants, dolphins, and rodents) indicates that polyproline length not only increases from amphibians to mammals but also varies significantly among mammals, perhaps in response to various functional loads. While neither the use of apatites nor the presence of proline-repeat polypeptides are unique for vertebrate mineralized tissues, vertebrates were nevertheless first in using polyproline repeat proteins to orchestrate the deposition of apatites into endoskeletal mineralized tissues. Microstructured apatites incorporated into innovative, highly flexible body plans not only gave these comparatively diminutive creatures a survival advantage over heavily armored organisms from the same period but also provided a mineral substrate for the evolution of teeth as powerful tools facilitating predation and food apprehension [44]–.

The ability of polyproline fragments alone to self-assemble and to guide apatite crystal growth in C-axis dimension raises the question about the role of the N-terminal and C-terminal amelogenin flanking domains. Here we propose that the flexible yet rigid structure of polyproline-rich assemblies provides a dynamic molecular packaging material between elongating mineral crystals. The evolution of elaborate mammalian enamel prisms as well as the design of the first vertebrate endoskeletons might thus be a result of sophisticated supramolecular polyproline matrices that insulate, guide, and package individual apatite crystals. Mirroring nature, the suitability of polyproline designer peptides to modulate apatite crystal growth emerges as a novel design concept for biomimetic enamel scaffolds and enamel tissue engineering.

## Materials and Methods

### Materials

Peptides (>99% purity) were synthesized by Genescript (Piscataway, NJ). The carbon coated copper TEM grids were purchased from SPI Supplies (West Chester, PA). Twelve mm coverslips were obtained from Fisher Scientific (Pittsburgh, PA). D_2_O (99.5%) was purchased from Cambridge Isotope Laboratories (Andover, MA). Four hundred MHz NMR tubes were obtained from Kontes (Vineland, NJ). Other common regents were from Sigma Aldrich (St Louis, MO).

### Cloning and Expression of Mouse Full-Length Amelogenin

The full-length mouse amelogenin coding sequence was cloned into pASK-43(+) with EcoR I and XhoI restriction site at 5′ and 3′ end, respectively. BL21-DM* was used as the host bacteria to express the recombinant proteins. The bacteria were cultured at 37°C until the OD_600_ reached 0.8 and then were induced at 32°C for 4 h. The expressed proteins were absorbed onto Ni-NTA agarose column and washed with 10 column volumes of PBS and 3 column volumes of 40 mM imidazole in PBS. Then the proteins were eluted with a pH 5.0 gradient (from 50 mM to 500 mM) imidazole PBS solution. The eluted proteins were dialyzed against H_2_O several times to make sure the salt and imidazole were diluted at least 10,000 times. Subsequently, the purified proteins were concentrated to about 10 mg/ml using a Centriprep YM-3 column. One litter bacteria culture yielded about 50 mg high quality mouse full-length amelogenin protein.

### Native Protein Extraction

Based on the high percentage of amelogenins in the enamel matrix of developing teeth, enamel from unerupted teeth was dissected and collected in 1 ml 6 M guanidine solution (pH 7.0) and incubated overnight to dissociate the enamel proteins from the enamel crystals. After centrifugation at 6,000 g for 15 min, the supernatant containing the amelogenin protein was dialyzed against water to remove the guanidine. Enamel proteins were then concentrated with YM-3 centricon columns.

### Scanning Electron Microscopy

Mice were sacrificed according to UIC animal care guidelines. For scanning electron microscopy, 20 d postnatal mouse mandibles were fixed in 4% paraformaldehyde and then saggitally hemisected using an Exakt sawing device. Enamel surfaces were etched in EDTA for 5 min, rinsed thoroughly, and dried overnight. Samples were coated with gold and palladium and then examined using a JEOL JSM-6320F scanning electron microscope. All measurements in this study were statistically evaluated using ANOVA and statistical dispersion was recorded and displayed using standard deviation (s.d.).

### HAP Crystal Growth

HAP crystal growth experiments were performed as previously described [21]. Briefly, peptides and proteins were dissolved in DDW at a concentration of 4 mg/ml and then adjusted to pH7.5–8.0 with 20 mM NH_4_OH at 4°C. Carbon coated copper TEM grids were immersed into the reaction mixture containing 1 mg/ml peptide/protein, 2.5 mM CaCl_2_, and 1.5 mM (NH_4_)_2_HPO_4_ and incubated in a moisturized container at 37°C for 2.5 h. Subsequently, TEM grids were quickly rinsed with DDW, blotted against filter paper, and air dried. Transmission electron microscopy was performed using a JEOL 1220 TEM. Electron diffraction patterns were collected as described earlier [29]. Briefly, patterns were obtained on 20 representative samples per group using a JEOL JEM-3010 in the diffraction mode at 300 kV and a camera length of 50 cm. Measurements were made at 90° incident to the sample. Patterns were measured for spot or ring diameter directly from the digital camera image, and the d spacings obtained were compared to those characteristic for hydroxyapatite.

### Peptide/Protein TEM Self-Assembly Experiments

Droplets containing 100 µl of diluted (1 mg/ml) pH7.5–8.0 peptide/protein solution were placed on carbon coated copper TEM grids and incubated in a moisturized container at 37°C for 2 h. Thereafter, TEM grids were quickly rinsed with DDW, immersed into 100 µl of freshly prepared 1% phosphotungstic acid solution for 6 min, quickly rinsed with DDW again, air dried, and analyzed using a Joel1220 TEM.

### Peptides/Proteins Self-Assembly Experiment for AFM

The AFM measurements were carried out using an extended MultiMode AFM (MMAFM) integrated with a NanoScope IIIa controller (Veeco Instruments, Santa Barbara, CA) and a Q-Control Module (nanoAnalytics, Muenster, Germany). The MMAFM was equipped with a calibrated E-type piezoelectric scanner and a glass cell for fluid TappingMode AFM (both from Veeco). The silicon AFM cantilever/probe used in this study was rectangular in shape, 130 µm in length and 35 µm in width (NSC36, MikroMasch). The advertised typical force constant and resonant frequency of this cantilever/probe is 0.6 N/m and 75 kHz, respectively. Nominal sharpness of the probe-tip end radius is ≤10 nm. The cantilever/probes were oscillated near 30 kHz at low amplitude for fluid tapping mode AFM. Fluid damping reduces the resonant frequency of rectangular AFM cantilevers in air by approximately 50%. The AFM substrate used for protein adsorption was Grade V5, Pelco mica (10×40 mm) purchased from Ted Pella (Redding, CA). The mica was freshly cleaved using adhesive tape prior to use. Stock solutions of 10–20 mg/ml protein in 40 mM Tris (pH 8.0) were mixed and stored at 4°C and analyzed by AFM within a few days. Stock solutions were diluted typically at 1∶100 into the blank AFM imaging buffer (40 mM Tris, pH 8.0) during scanning and adsorption to mica was monitored. Typical AFM scan rates were 1.0–1.25 Hz for 512 data points×256 lines. The AFM images were planefit to correct for background sloping errors.

### Transgenic Mice Overexpressing the *Rana pipiens* Amelogenin Gene

The mouse amelogenin genomic fragment was obtained by PCR amplification of the BAC clone RP23-334F21 (X-chromosome), containing the amelogenin promoter region. We amplified −2.3 kb of a region that included the promoter, exon 1, intron 1, and part of exon 2. Primers 1 and 2 (Figure S1) were used to amplify a region from the ApaI (−2,345) site to the EcoRI (−262) site and Primers 3 and 4 to amplify a region from the EcoRI (−262) site to the ATG start codon on exon 2 including the mouse amelogenin signal peptide region. Primers 5 and 6 amplified a fragment that ranged from the first amino acids of the frog amelogenin to the stop codon based on our frog amelogenin cDNA plasmid [35]. All three fragments were cloned into the pBSKII modified vector (Stratagene, La Jolla, CA) containing poly A. For cross-breeding studies, we mated mouse homozygous amelogenin knockout mice with over-expressing frog amelogenin transgenic mice. These mouse amelogenin knockout and frog amelogenin over-expressing compound mice were used to study frog amelogenin function in vivo. For further analysis, enamel of first mandibular molar from five amelogenin null, fAmel-x-null, and wild-type mice each was analyzed and compared between groups. The phenotype in fAmel mice alone was less severe and thus not used for further analysis.

### Nanosphere and Mineral Crystal Measurement

Following sample processing for electron microscopy, 20 electron micrographs per sample from each group were collected and further processed for image analysis. Crystal dimensions were converted from pixels into nanometers based on electron micrograph reference bars. For nanosphere measurements, 5 micrographs were measured and at least 30 nanospheres in each micrographs were selected. For the mineral crystals measurements, 5 micrographs were measured and at least 10 crystal needles in each micrograph were selected. All the data were analyzed with SPSS software using the ANOVA test.

### Nuclear Magnetic Resonance

All NMR measurements were performed in either 10% D_2_O/90% H_2_O or 100% D2O at 10°C on a Bruker DRX 800MHz spectrometer. The concentration of individual peptides was 5 mg/ml. Standard homonuclear 2D TOCSY, NOESY, and COSY experiments were conducted in order to generate backbone, side chain, and NOE constraint assignments. The mixing time for TOCSY and NOESY was 80 ms and 150 ms, respectively. ^13^C-HSQC was performed with the naturally abundant ^13^C isotope. Spectra were processed and analyzed using the SYBYL software package (Tripos, MO). All 1H dimensions were referenced to internal 2,2-dimethyl-2-silapentane-5-sulfinate (DSS). NOE constraints were manually classified into strong (2Å), medium (4Å), and weak (6Å) groups. The sequence-specific backbone resonance assignment was achieved through a combination of 2D NOESY, TOCSY, and ^13^C-HSQC spectra by matching chemical shifts for a given residue or short distance NOE signals. Structure calculations were performed with the DYANA 1.5 program [47], using a 40,000-step energy minimization procedure. All subsequent analyses of the structure and graphic representations of the three-dimensional structures were performed using MolMol [48].

## Supporting Information

Figure S1 ***Rana pipiens*** **Amelogenin expressing transgenic mouse construct.**
(0.08 MB DOC)

Table S1 **PXX33 chemical shift table.**
(0.08 MB DOC)

Table S2 **Detected NOEs of the PXX33 peptide.**
(0.33 MB DOC)

## Abbreviations

AFM: atomic force microscopy
CD: circular dichroism
DLS: dynamic light scattering
EMSP1: Enamel matrix serine proteinase 1
fAmel-x-null mice: *Rana pipiens* amelogenin expressing mice
MMAFM: MultiMode AFM
MMP20: matrix metalloproteinase 20
NOE: Nuclear Overhauser Effect
NOESY: Nuclear Overhauser Effect Spectroscopy
PPII: polyproline II
PRAP: proline-rich amelogenin peptide
RMSD: root mean square deviation
TEM: transmission electron micrographs
