# Supplementary figures and images for: Elongated Polyproline Motifs Facilitate Enamel Evolution through Matrix Subunit Compaction
Source: PLoS Biol. 2009 Dec 22;7(12):e1000262. doi: 10.1371/journal.pbio.1000262 (PMC2787623; doi:10.1371/journal.pbio.1000262)

**Figure S1** *Rana pipiens* Amelogenin expressing transgenic mouse construct.


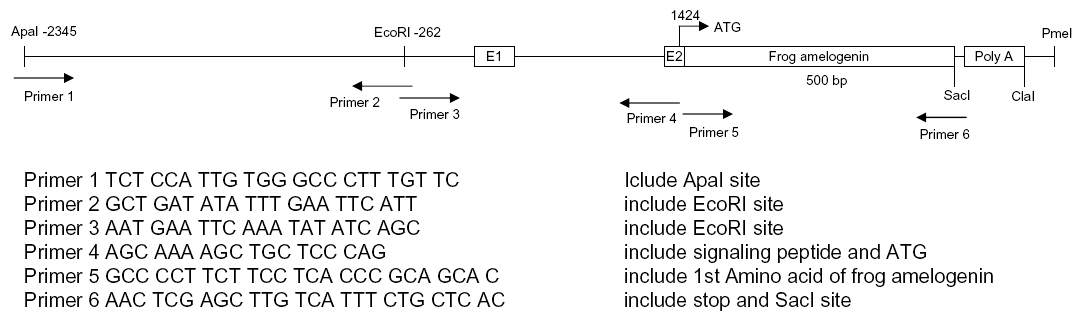

Supplement: Figure S1 — Rana pipiens Amelogenin expressing transgenic mouse construct. (0.08 MB DOC) [file pbio.1000262.s001.doc]
