# Supplementary material for: Elongated Polyproline Motifs Facilitate Enamel Evolution through Matrix Subunit Compaction
Source: PLoS Biol. 2009 Dec 22;7(12):e1000262. doi: 10.1371/journal.pbio.1000262 (PMC2787623; doi:10.1371/journal.pbio.1000262)
Supplement: Table S1 — PXX33 chemical shift table. (0.08 MB DOC) [file pbio.1000262.s002.doc]

# Table S1 PXX33 Chemical shift table.

|  | NH | Hα | Hβ1 | Hβ2 | Hγ1 | Hγ2 | Hδ1 | Hδ2 | Hε1 | Hε2 |
| --- | --- | --- | --- | --- | --- | --- | --- | --- | --- | --- |
| P1 |  |  |  |  |  |  |  |  |  |  |
| M2 | 8.81 | 4.1204 | 1.7649 | 2.174 | 3.098 | 3.142 |  |  |  |  |
| Q3 | 8.605 | 4.188 | 1.7267 | 1.7721 | 2.286 | 2.32 |  |  |  |  |
| P4 |  | 4.138 | 2.005 |  |  |  | 3.361 | 3.532 |  |  |
| Q5 | 8.37 | 4.306 | 1.620 | 1.804 | 2.146 |  |  |  |  |  |
| P6 |  | 4.428 |  |  |  |  | 3.794 | 3.757 |  |  |
| P7 |  | 4.146 | 1.994 |  | 1.5176 |  | 3.361 | 3.532 |  |  |
| V8 | 8.044 | 3.696 | 1.6741 |  | 0.630 | 0.560 |  |  |  |  |
| H9 | 8.3782 | 4.749 | 2.856 | 2.9545 |  |  |  |  | 7.034 | 8.348 |
| P10 |  | 4.003 | 1.9873 | 2.1444 | 1.7161 | 1.6166 | 3.3223 | 3.5029 |  |  |
| M11 | 8.434 | 4.172 | 1.73 | 1.774 | 2.29 | 2.342 |  |  |  |  |
| Q12 | 8.166 | 4.124 | 1.7649 | 2.172 | 3.098 | 3.142 |  |  |  |  |
| Q17 | 8.424 |  |  |  |  |  |  |  |  |  |
| P18 |  |  |  |  |  |  |  |  |  |  |
| P19? |  | 4.411 | 1.986 | 2.262 | 1.7366 | 1.6048 | 3.356 | 3.524 |  |  |
| L20 | 8.29 | 4.137 | 1.736 | 1.774 | 1.384 | 1.540 | 0.662 |  |  |  |
| P21 |  |  |  |  |  |  |  |  |  |  |
| P22 |  | 4.067 | 1.946 | 1.727 | 1.488 | 1.490 | 3.512 | 3.342 |  |  |
| M23 | 8.130 | 4.0313 | 1.60 | 1.740 | 2.078 | 2.187 |  |  |  |  |
| F24 | 8.01 | 4.629 | 2.628 | 2.882 |  |  | 7.01 | 7.07 | 6.983 | 7.102 |
| P25 |  |  | 1.976 |  | 1.708 |  | 3.296 | 3.473 |  |  |
| M26 | 8.290 | 4.137 | 1.736 | 1.774 | 1.384 |  |  |  |  |  |
| Q27 | 8.247 | 4.166 | 1.748 | 1.81 | 2.3 | 2.35 |  |  |  |  |
| P28 |  | 4.404 |  | 1.984 |  |  |  |  |  |  |
| L29 | 8.1448 | 4.294 | 2.189 | 1.758 | 1.278 | 1.432 |  | 0.627 | 0.642 |  |
| P30 |  |  |  |  |  |  |  |  |  |  |
| P31 |  |  |  |  |  |  | 3.248 | 3.592 |  |  |
| M32 | 8.44 | 4.347 | 1.814 | 2.14 | 1.670 |  |  |  |  |  |
| L33 | 8.229 | 4.324 | 1.664 | 2.126 | 1.284 | 1.448 | 0.662 |  |  |  |
