# Supplementary material for: Elongated Polyproline Motifs Facilitate Enamel Evolution through Matrix Subunit Compaction
Source: PLoS Biol. 2009 Dec 22;7(12):e1000262. doi: 10.1371/journal.pbio.1000262 (PMC2787623; doi:10.1371/journal.pbio.1000262)
Supplement: Table S2 — Detected NOEs of the PXX33 peptide. (0.33 MB DOC) [file pbio.1000262.s003.doc]

**Table S2** Detected NOEs of the PXX33 peptide.

| Position | residue | atom | Position | residue | atom | distance |
| --- | --- | --- | --- | --- | --- | --- |
| 2 | MET | HA | 3 | GLN | HN | 3 |
| 2 | MET | HB2 | 3 | GLN | HN | 4.5 |
| 2 | MET | HG3 | 3 | GLN | HN | 5.5 |
| 2 | MET | HG2 | 3 | GLN | HN | 5.5 |
| 3 | GLN | HN | 2 | MET | HN | 5.5 |
| 5 | GLN | HN | 7 | PRO | HD2 | 4.5 |
| 5 | GLN | HN | 4 | PRO | HB3 | 4.5 |
| 5 | GLN | HN | 4 | PRO | HA | 3 |
| 7 | PRO | HA | 8 | VAL | HN | 3.5 |
| 7 | PRO | QB | 8 | VAL | HN | 3 |
| 7 | PRO | QB | 9 | HIS | HB3 | 7.5 |
| 7 | PRO | QG | 8 | VAL | HN | 3 |
| 7 | PRO | QG | 8 | VAL | HA | 5.4 |
| 7 | PRO | HD3 | 8 | VAL | HN | 4.5 |
| 7 | PRO | HD3 | 5 | GLN | HA | 5.5 |
| 7 | PRO | HD2 | 8 | VAL | HN | 4.5 |
| 7 | PRO | HD2 | 5 | GLN | HA | 3 |
| 8 | VAL | HN | 9 | HIS | HN | 3 |
| 8 | VAL | HN | 9 | HIS | HA | 5.5 |
| 8 | VAL | HN | 9 | HIS | HB3 | 5.5 |
| 8 | VAL | HN | 9 | HIS | HB2 | 5.9 |
| 8 | VAL | HN | 9 | HIS | HD2 | 5.5 |
| 8 | VAL | HA | 9 | HIS | HN | 3.6 |
| 8 | VAL | HA | 9 | HIS | HA | 5 |
| 8 | VAL | HA | 9 | HIS | HB3 | 5 |
| 8 | VAL | HA | 9 | HIS | HB2 | 6.1 |
| 8 | VAL | HA | 9 | HIS | HD2 | 4.5 |
| 8 | VAL | HA | 7 | PRO | HD2 | 5.5 |
| 8 | VAL | HA | 7 | PRO | QB | 5.5 |
| 8 | VAL | HB | 9 | HIS | HN | 4.5 |
| 8 | VAL | HB | 9 | HIS | HD2 | 5.5 |
| 8 | VAL | QG1 | 9 | HIS | HN | 3 |
| 8 | VAL | QG1 | 9 | HIS | HD2 | 5.5 |
| 8 | VAL | QG2 | 9 | HIS | HN | 4.5 |
| 8 | VAL | QG2 | 9 | HIS | HD2 | 5.5 |
| 8 | VAL | QG2 | 9 | HIS | HB3 | 4.5 |
| 8 | VAL | QG2 | 9 | HIS | HB2 | 5.5 |
| 8 | VAL | QG2 | 10 | PRO | HD2 | 7 |
| 9 | HIS | HN | 9 | HIS | HD2 | 4.5 |
| 9 | HIS | HN | 10 | PRO | HA | 5 |
| 9 | HIS | HN | 10 | PRO | HD3 | 5 |
| 9 | HIS | HN | 10 | PRO | HD2 | 5 |
| 9 | HIS | HN | 7 | PRO | QG | 4.8 |
| 9 | HIS | HA | 9 | HIS | HD2 | 5.5 |
| 9 | HIS | HA | 10 | PRO | HD3 | 3.5 |
| 9 | HIS | HA | 10 | PRO | HD2 | 4 |
| 9 | HIS | HA | 11 | MET | HN | 4.5 |
| 9 | HIS | HB3 | 9 | HIS | HD1 | 5.5 |
| 9 | HIS | HB3 | 9 | HIS | HD2 | 4.5 |
| 9 | HIS | HB3 | 8 | VAL | HN | 5.5 |
| 9 | HIS | HB3 | 10 | PRO | HD3 | 3.5 |
| 9 | HIS | HB3 | 10 | PRO | HD2 | 3.5 |
| 9 | HIS | HB3 | 11 | MET | HN | 5 |
| 9 | HIS | HB2 | 9 | HIS | HD1 | 5.5 |
| 9 | HIS | HB2 | 9 | HIS | HD2 | 4.5 |
| 9 | HIS | HB2 | 10 | PRO | HD3 | 3 |
| 9 | HIS | HB2 | 10 | PRO | HD2 | 3 |
| 9 | HIS | HB2 | 11 | MET | HN | 4.5 |
| 9 | HIS | HD2 | 9 | HIS | HD1 | 4.5 |
| 9 | HIS | HD2 | 9 | HIS | HA | 5.5 |
| 9 | HIS | HD2 | 9 | HIS | HB3 | 4.5 |
| 9 | HIS | HD2 | 9 | HIS | HB2 | 4.5 |
| 9 | HIS | HD2 | 7 | PRO | QB | 5.5 |
| 9 | HIS | HD1 | 8 | VAL | QG2 | 4.5 |
| 10 | PRO | HA | 9 | HIS | HB3 | 6 |
| 10 | PRO | HA | 9 | HIS | HB2 | 5.5 |
| 10 | PRO | HD3 | 11 | MET | HN | 4.5 |
| 10 | PRO | HD2 | 11 | MET | HN | 4.5 |
| 11 | MET | HN | 12 | GLN | HN | 3.5 |
| 11 | MET | HN | 9 | HIS | HD2 | 5.5 |
| 11 | MET | HN | 10 | PRO | HB3 | 5 |
| 11 | MET | HN | 8 | VAL | QG1 | 5.5 |
| 11 | MET | HN | 8 | VAL | QG2 | 6 |
| 12 | GLN | HN | 11 | MET | HG2 | 5.5 |
| 20 | LEU | HN | 19 | PRO | HD2 | 4.5 |
| 20 | LEU | HN | 19 | PRO | HA | 4.5 |
| 20 | LEU | HN | 18 | PRO | HD2 | 5.5 |
| 22 | PRO | HB3 | 23 | MET | HN | 5.5 |
| 23 | MET | HN | 24 | PHE | HN | 3 |
| 23 | MET | HN | 24 | PHE | HA | 4.5 |
| 23 | MET | HN | 24 | PHE | HB3 | 5 |
| 23 | MET | HN | 24 | PHE | HB2 | 5.5 |
| 23 | MET | HN | 24 | PHE | HD2 | 4.5 |
| 23 | MET | HN | 24 | PHE | HD1 | 5 |
| 23 | MET | HN | 22 | PRO | HA | 4.5 |
| 23 | MET | HN | 22 | PRO | HD3 | 4.5 |
| 23 | MET | HN | 22 | PRO | HD2 | 4.5 |
| 23 | MET | HN | 22 | PRO | HB2 | 4.5 |
| 23 | MET | HN | 22 | PRO | QG | 4.5 |
| 23 | MET | HA | 24 | PHE | HN | 3 |
| 23 | MET | HA | 24 | PHE | HB3 | 5.5 |
| 23 | MET | HG3 | 24 | PHE | HB3 | 4.5 |
| 23 | MET | HG3 | 24 | PHE | HB2 | 6 |
| 24 | PHE | HN | 24 | PHE | HD2 | 4.5 |
| 24 | PHE | HN | 23 | MET | HG2 | 5.5 |
| 24 | PHE | HN | 23 | MET | HG3 | 4.5 |
| 24 | PHE | HN | 23 | MET | HB3 | 4.5 |
| 24 | PHE | HN | 23 | MET | HB2 | 4.5 |
| 24 | PHE | HN | 25 | PRO | HD3 | 4.5 |
| 24 | PHE | HN | 25 | PRO | HD2 | 5 |
| 24 | PHE | HN | 22 | PRO | QG | 4.5 |
| 24 | PHE | HN | 22 | PRO | HB2 | 4.5 |
| 24 | PHE | HA | 24 | PHE | HD2 | 4.5 |
| 24 | PHE | HA | 23 | MET | HB3 | 5.5 |
| 24 | PHE | HA | 25 | PRO | HD3 | 4.5 |
| 24 | PHE | HA | 25 | PRO | HD2 | 4.5 |
| 24 | PHE | HA | 25 | PRO | QG | 5.5 |
| 24 | PHE | HA | 28 | PRO | QB | 5.5 |
| 24 | PHE | HA | 26 | MET | HN | 5.5 |
| 24 | PHE | HB3 | 23 | MET | HB3 | 5.5 |
| 24 | PHE | HB3 | 25 | PRO | HD3 | 4.5 |
| 24 | PHE | HB3 | 25 | PRO | HD2 | 4.5 |
| 24 | PHE | HB3 | 25 | PRO | QG | 6.5 |
| 24 | PHE | HB2 | 24 | PHE | HD2 | 4.5 |
| 24 | PHE | HB2 | 25 | PRO | HD3 | 4.5 |
| 24 | PHE | HB2 | 25 | PRO | HD2 | 4.5 |
| 24 | PHE | HB2 | 25 | PRO | QG | 5.5 |
| 24 | PHE | HB2 | 28 | PRO | QB | 5.5 |
| 24 | PHE | HD2 | 24 | PHE | HN | 4.5 |
| 24 | PHE | HD2 | 24 | PHE | HA | 4.5 |
| 24 | PHE | HD2 | 24 | PHE | HB3 | 4 |
| 24 | PHE | HD2 | 24 | PHE | HB2 | 3 |
| 24 | PHE | HD2 | 23 | MET | HB3 | 5.5 |
| 24 | PHE | HD2 | 25 | PRO | HA | 4.5 |
| 24 | PHE | HD2 | 25 | PRO | HD3 | 5 |
| 24 | PHE | HD2 | 25 | PRO | HD2 | 4.5 |
| 24 | PHE | HD2 | 22 | PRO | QG | 5.5 |
| 24 | PHE | HD1 | 24 | PHE | HA | 5.5 |
| 24 | PHE | HD1 | 24 | PHE | HB3 | 5.5 |
| 24 | PHE | HD1 | 24 | PHE | HB2 | 5.5 |
| 24 | PHE | HD1 | 23 | MET | HA | 5.5 |
| 24 | PHE | HD1 | 23 | MET | HG3 | 5.5 |
| 24 | PHE | HD1 | 23 | MET | HB3 | 5.5 |
| 26 | MET | HN | 27 | GLN | HN | 5.5 |
| 26 | MET | HN | 23 | MET | HN | 5.5 |
| 26 | MET | HN | 24 | PHE | HN | 5.5 |
| 26 | MET | HN | 24 | PHE | HA | 4.5 |
| 26 | MET | HN | 24 | PHE | HD2 | 5.5 |
| 26 | MET | HN | 24 | PHE | HD1 | 5.5 |
| 26 | MET | HN | 25 | PRO | HD2 | 4.5 |
| 26 | MET | HN | 25 | PRO | HD3 | 4.5 |
| 26 | MET | HN | 28 | PRO | HD2 | 5.5 |
| 26 | MET | HN | 28 | PRO | HD3 | 4.5 |
| 27 | GLN | HN | 24 | PHE | HD1 | 6 |
| 27 | GLN | HN | 24 | PHE | HE2 | 5 |
| 27 | GLN | HN | 28 | PRO | HD2 | 5.5 |
| 27 | GLN | HN | 28 | PRO | HD3 | 4.5 |
| 29 | LEU | HN | 28 | PRO | HA | 5.5 |
| 32 | MET | HN | 33 | LEU | HN | 3 |
| 33 | LEU | HN | 31 | PRO | HD2 | 4.5 |
| 33 | LEU | HN | 31 | PRO | HD3 | 4.5 |
